# Bis(2-benzyl­imino­methyl-4,6-dihydro­seleno­phenolato)iron(II)

**DOI:** 10.1107/S1600536809032413

**Published:** 2009-08-22

**Authors:** Hai-Yan Li, Li-Jun Wang, Jian Hou, Qing-Fu Zeng

**Affiliations:** a Engineering Research Center for Clean Production of Textile Dyeing and Printing, Ministry of Education, Wuhan 430073, People’s Republic of China

## Abstract

In the title compound, [Fe(C_14_H_12_NOSe_2_)_2_], the Fe^II^ ion (site symmetry 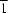) is four-coordinated by two *N*,*O*-bidentate Schiff base ligands, resulting in a slightly distorted *trans*-FeN_2_O_2_ square-planar coordination for the metal ion.

## Related literature

For background, see: Shi *et al.* (2008 ▶); Xu *et al.* (2009 ▶). For reference structural data, see: Allen *et al.* (1987 ▶).
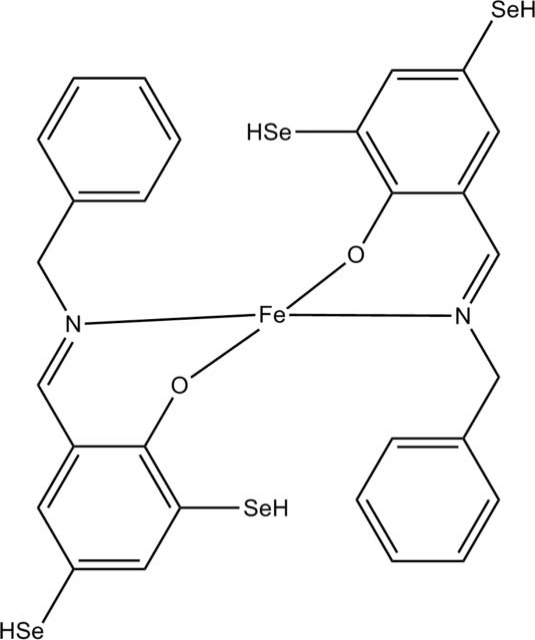


## Experimental

### 

#### Crystal data

[Fe(C_14_H_12_NOSe_2_)_2_]
*M* *_r_* = 792.18
Monoclinic, 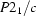

*a* = 10.6065 (5) Å
*b* = 6.1055 (5) Å
*c* = 20.7125 (15) Å
β = 102.435 (5)°
*V* = 1309.83 (16) Å^3^
*Z* = 2
Mo *K*α radiation
μ = 6.16 mm^−1^
*T* = 296 K
0.32 × 0.26 × 0.24 mm

#### Data collection

Enraf–Nonius CAD-4 diffractometer
Absorption correction: ψ scan (North *et al.*, 1968 ▶) *T*_min_ = 0.243, *T*_max_ = 0.319 (expected range = 0.174–0.228)
8208 measured reflections
3231 independent reflections
2133 reflections with *I* > 2σ(*I*)
*R*_int_ = 0.044
3 standard reflections every 200 reflections intensity decay: 1%

#### Refinement

*R*[*F* ^2^ > 2σ(*F* ^2^)] = 0.040
*wR*(*F* ^2^) = 0.105
*S* = 1.05
3231 reflections
175 parameters
2 restraints
H atoms treated by a mixture of independent and constrained refinement
Δρ_max_ = 0.68 e Å^−3^
Δρ_min_ = −0.61 e Å^−3^

### 

Data collection: *CAD-4 Software* (Enraf–Nonius, 1989 ▶); cell refinement: *CAD-4 Software*; data reduction: *XCAD4* (Harms & Wocadlo, 1995 ▶); program(s) used to solve structure: *SHELXS97* (Sheldrick, 2008 ▶); program(s) used to refine structure: *SHELXL97* (Sheldrick, 2008 ▶); molecular graphics: *SHELXTL* (Sheldrick, 2008 ▶); software used to prepare material for publication: *SHELXTL*.

## Supplementary Material

Crystal structure: contains datablocks global, I. DOI: 10.1107/S1600536809032413/hb5048sup1.cif

Structure factors: contains datablocks I. DOI: 10.1107/S1600536809032413/hb5048Isup2.hkl

Additional supplementary materials:  crystallographic information; 3D view; checkCIF report

## Figures and Tables

**Table:** 

Fe1—O1	1.845 (2)
Fe1—N1	1.945 (3)

**Table:** 

O1—Fe1—N1	91.85 (12)

**Fig. 1.**
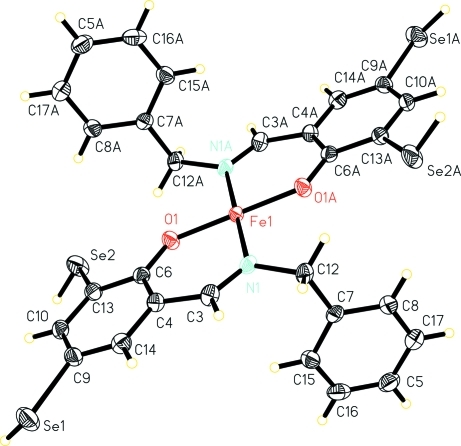
The molecular structure of (I) showing 30% probability displacement ellipsoids, Atoms with an A suffix to their labels are generated by the symmetry operation (1–x, 1–y, –z).

## supplementary crystallographic information

### Comment

There has been much research interest in Schiff base metal complexes due to their molecular architectures and biological activities (Shi *et al.*, 2008; Xu *et al.*, 2009). In this work, we report here the crystal structure of the title compound, (I). In (I), all bond lengths are within normal ranges (Allen *et al.*, 1987) (Fig. 1). The Fe^II^ is four-coordinated in a slightly distort square-planar configuration by two N atoms and two O atoms of the Schiff base ligands.

### Experimental

A mixture of 3,5-dihydroseleno-2-hydroxybenzaldehyde (564 mg, 2 mmol), phenylmethanamine (107 mg, 2 mmol) and FeCl_2_.4H_2_O (1 mmol, 198 mg) in methanol (10 ml) was stirred for 1 h. After keeping the filtrate in air for 7 d, green blocks of (I) were formed.

### Refinement

All H atoms were positioned geometrically (C—H = 0.93 Å for the aromatic H atoms and C—H = 0.96 Å for the aliphatic H atoms) and were refined as riding, with *U*_iso_(H) = 1.2*U*_eq_(C) and *U*_iso_(H) = 1.2*U*_eq_(N).

### Crystal data

**Table:**

[Fe(C_14_H_12_NOSe_2_)_2_]	*F*(000) = 768
*M**_r_* = 792.18	*D*_x_ = 2.009 Mg m^−^^3^
Monoclinic, *P*2_1_/*c*	Mo *K*α radiation, λ = 0.71073 Å
Hall symbol: -P 2ybc	Cell parameters from 25 reflections
*a* = 10.6065 (5) Å	θ = 9–12°
*b* = 6.1055 (5) Å	µ = 6.16 mm^−^^1^
*c* = 20.7125 (15) Å	*T* = 296 K
β = 102.435 (5)°	Block, green
*V* = 1309.83 (16) Å^3^	0.32 × 0.26 × 0.24 mm
*Z* = 2	

### Data collection

**Table:**

Enraf–Nonius CAD-4 diffractometer	2133 reflections with *I* > 2σ(*I*)
Radiation source: fine-focus sealed tube	*R*_int_ = 0.044
graphite	θ_max_ = 28.6°, θ_min_ = 2.0°
ω/2θ scans	*h* = −13→12
Absorption correction: ψ scan (North *et al.*, 1968)	*k* = −8→8
*T*_min_ = 0.243, *T*_max_ = 0.319	*l* = −27→24
8208 measured reflections	3 standard reflections every 200 reflections
3231 independent reflections	intensity decay: 1%

### Refinement

**Table:**

Refinement on *F*^2^	Primary atom site location: structure-invariant direct methods
Least-squares matrix: full	Secondary atom site location: difference Fourier map
*R*[*F*^2^ > 2σ(*F*^2^)] = 0.040	Hydrogen site location: inferred from neighbouring sites
*wR*(*F*^2^) = 0.105	H atoms treated by a mixture of independent and constrained refinement
*S* = 1.05	*w* = 1/[σ^2^(*F*_o_^2^) + (0.0464*P*)^2^] where *P* = (*F*_o_^2^ + 2*F*_c_^2^)/3
3231 reflections	(Δ/σ)_max_ = 0.015
175 parameters	Δρ_max_ = 0.68 e Å^−^^3^
2 restraints	Δρ_min_ = −0.61 e Å^−^^3^

### Special details

**Table:**

Geometry. All e.s.d.'s (except the e.s.d. in the dihedral angle between two l.s. planes) are estimated using the full covariance matrix. The cell e.s.d.'s are taken into account individually in the estimation of e.s.d.'s in distances, angles and torsion angles; correlations between e.s.d.'s in cell parameters are only used when they are defined by crystal symmetry. An approximate (isotropic) treatment of cell e.s.d.'s is used for estimating e.s.d.'s involving l.s. planes.
Refinement. Refinement of *F*^2^ against ALL reflections. The weighted *R*-factor *wR* and goodness of fit *S* are based on *F*^2^, conventional *R*-factors *R* are based on *F*, with *F* set to zero for negative *F*^2^. The threshold expression of *F*^2^ > σ(*F*^2^) is used only for calculating *R*-factors(gt) *etc*. and is not relevant to the choice of reflections for refinement. *R*-factors based on *F*^2^ are statistically about twice as large as those based on *F*, and *R*- factors based on ALL data will be even larger.

### Fractional atomic coordinates and isotropic or equivalent isotropic displacement parameters (Å^2^)

**Table:**

	*x*	*y*	*z*	*U*_iso_*/*U*_eq_	
Fe1	0.5000	0.5000	0.0000	0.02627 (19)	
Se1	0.39624 (5)	1.46692 (8)	0.19627 (3)	0.06046 (19)	
Se2	0.11313 (4)	0.76022 (9)	0.06333 (3)	0.05734 (19)	
N1	0.6354 (3)	0.6850 (5)	0.05035 (16)	0.0338 (7)	
O1	0.3733 (2)	0.6579 (5)	0.02791 (13)	0.0383 (7)	
C3	0.6153 (4)	0.8701 (7)	0.0771 (2)	0.0370 (9)	
H3	0.6877	0.9562	0.0930	0.044*	
C4	0.4943 (3)	0.9565 (6)	0.08496 (19)	0.0333 (9)	
C5	0.8814 (4)	0.1903 (9)	0.2276 (2)	0.0519 (12)	
H5	0.9044	0.0960	0.2635	0.062*	
C6	0.3792 (3)	0.8364 (6)	0.06265 (18)	0.0322 (9)	
C7	0.8111 (3)	0.4726 (6)	0.12025 (19)	0.0339 (9)	
C8	0.8905 (4)	0.2934 (7)	0.1180 (2)	0.0433 (10)	
H8	0.9206	0.2664	0.0798	0.052*	
C9	0.3884 (4)	1.2227 (6)	0.1388 (2)	0.0393 (9)	
C10	0.2727 (4)	1.1107 (7)	0.11830 (19)	0.0403 (10)	
H10	0.1978	1.1635	0.1293	0.048*	
C12	0.7746 (3)	0.6234 (7)	0.0611 (2)	0.0359 (9)	
H12A	0.8275	0.7544	0.0684	0.043*	
H12B	0.7905	0.5504	0.0220	0.043*	
C13	0.2689 (4)	0.9232 (7)	0.08202 (19)	0.0370 (9)	
C14	0.4963 (4)	1.1457 (6)	0.1226 (2)	0.0398 (10)	
H14	0.5737	1.2204	0.1368	0.048*	
C15	0.7687 (4)	0.5074 (7)	0.1783 (2)	0.0425 (10)	
H15	0.7167	0.6274	0.1820	0.051*	
C16	0.8040 (4)	0.3632 (8)	0.2308 (2)	0.0504 (12)	
H16	0.7734	0.3864	0.2691	0.061*	
C17	0.9257 (4)	0.1547 (8)	0.1713 (2)	0.0498 (11)	
H17	0.9798	0.0364	0.1689	0.060*	
H1	0.318 (3)	1.461 (7)	0.2300 (18)	0.060*	
H2	0.064 (4)	0.795 (7)	0.1091 (14)	0.060*	

### Atomic displacement parameters (Å^2^)

**Table:**

	*U*^11^	*U*^22^	*U*^33^	*U*^12^	*U*^13^	*U*^23^
Fe1	0.0184 (3)	0.0306 (4)	0.0295 (4)	−0.0017 (3)	0.0046 (3)	−0.0018 (3)
Se1	0.0802 (4)	0.0432 (3)	0.0599 (4)	0.0061 (2)	0.0195 (3)	−0.0142 (2)
Se2	0.0286 (2)	0.0838 (4)	0.0631 (4)	−0.0073 (2)	0.0177 (2)	−0.0260 (3)
N1	0.0228 (15)	0.0406 (19)	0.0364 (19)	0.0003 (13)	0.0028 (13)	0.0025 (15)
O1	0.0262 (13)	0.0458 (17)	0.0436 (17)	−0.0017 (11)	0.0092 (12)	−0.0112 (14)
C3	0.0283 (19)	0.035 (2)	0.047 (3)	−0.0040 (16)	0.0069 (18)	0.004 (2)
C4	0.0310 (19)	0.032 (2)	0.037 (2)	−0.0006 (15)	0.0079 (17)	0.0007 (18)
C5	0.047 (3)	0.066 (3)	0.039 (3)	0.003 (2)	0.000 (2)	0.006 (2)
C6	0.0314 (19)	0.035 (2)	0.030 (2)	0.0012 (16)	0.0070 (16)	0.0023 (18)
C7	0.0201 (16)	0.044 (2)	0.035 (2)	−0.0050 (16)	−0.0009 (15)	−0.0045 (18)
C8	0.037 (2)	0.055 (3)	0.038 (2)	0.0061 (19)	0.0076 (18)	−0.005 (2)
C9	0.051 (2)	0.034 (2)	0.035 (2)	0.0071 (18)	0.0113 (19)	0.0010 (18)
C10	0.040 (2)	0.048 (3)	0.035 (2)	0.0138 (19)	0.0132 (19)	0.006 (2)
C12	0.0211 (17)	0.041 (2)	0.046 (2)	−0.0033 (15)	0.0070 (17)	−0.0012 (19)
C13	0.033 (2)	0.046 (3)	0.034 (2)	0.0039 (17)	0.0106 (17)	0.0013 (19)
C14	0.042 (2)	0.034 (2)	0.043 (2)	−0.0020 (18)	0.0059 (19)	0.003 (2)
C15	0.040 (2)	0.046 (3)	0.041 (3)	0.0009 (19)	0.0073 (19)	−0.011 (2)
C16	0.048 (3)	0.072 (3)	0.030 (2)	−0.009 (2)	0.007 (2)	−0.005 (2)
C17	0.046 (2)	0.054 (3)	0.046 (3)	0.010 (2)	0.003 (2)	0.003 (2)

### Geometric parameters (Å, °)

**Table:**

Fe1—O1	1.845 (2)	C6—C13	1.419 (5)
Fe1—O1^i^	1.845 (2)	C7—C8	1.387 (5)
Fe1—N1^i^	1.945 (3)	C7—C15	1.388 (5)
Fe1—N1	1.945 (3)	C7—C12	1.516 (5)
Se1—C9	1.898 (4)	C8—C17	1.378 (6)
Se1—H1	1.191 (10)	C8—H8	0.9300
Se2—C13	1.896 (4)	C9—C14	1.345 (5)
Se2—H2	1.196 (10)	C9—C10	1.390 (6)
N1—C3	1.296 (5)	C10—C13	1.365 (6)
N1—C12	1.493 (4)	C10—H10	0.9300
O1—C6	1.300 (4)	C12—H12A	0.9700
C3—C4	1.429 (5)	C12—H12B	0.9700
C3—H3	0.9300	C14—H14	0.9300
C4—C14	1.391 (5)	C15—C16	1.387 (6)
C4—C6	1.414 (5)	C15—H15	0.9300
C5—C16	1.348 (6)	C16—H16	0.9300
C5—C17	1.366 (6)	C17—H17	0.9300
C5—H5	0.9300		
			
O1—Fe1—O1^i^	180.0	C7—C8—H8	119.3
O1—Fe1—N1^i^	88.15 (12)	C14—C9—C10	119.6 (4)
O1^i^—Fe1—N1^i^	91.85 (12)	C14—C9—Se1	120.5 (3)
O1—Fe1—N1	91.85 (12)	C10—C9—Se1	119.7 (3)
O1^i^—Fe1—N1	88.15 (12)	C13—C10—C9	120.1 (4)
N1^i^—Fe1—N1	180.0	C13—C10—H10	120.0
C9—Se1—H1	114 (2)	C9—C10—H10	120.0
C13—Se2—H2	105 (2)	N1—C12—C7	110.2 (3)
C3—N1—C12	113.9 (3)	N1—C12—H12A	109.6
C3—N1—Fe1	124.4 (3)	C7—C12—H12A	109.6
C12—N1—Fe1	121.7 (3)	N1—C12—H12B	109.6
C6—O1—Fe1	131.3 (2)	C7—C12—H12B	109.6
N1—C3—C4	127.2 (4)	H12A—C12—H12B	108.1
N1—C3—H3	116.4	C10—C13—C6	122.7 (4)
C4—C3—H3	116.4	C10—C13—Se2	118.3 (3)
C14—C4—C6	121.4 (3)	C6—C13—Se2	118.8 (3)
C14—C4—C3	117.7 (3)	C9—C14—C4	121.3 (4)
C6—C4—C3	120.4 (3)	C9—C14—H14	119.4
C16—C5—C17	119.4 (4)	C4—C14—H14	119.4
C16—C5—H5	120.3	C16—C15—C7	119.9 (4)
C17—C5—H5	120.3	C16—C15—H15	120.0
O1—C6—C4	123.4 (3)	C7—C15—H15	120.0
O1—C6—C13	121.7 (3)	C5—C16—C15	121.6 (4)
C4—C6—C13	114.9 (4)	C5—C16—H16	119.2
C8—C7—C15	117.5 (4)	C15—C16—H16	119.2
C8—C7—C12	120.2 (3)	C5—C17—C8	120.2 (4)
C15—C7—C12	122.2 (3)	C5—C17—H17	119.9
C17—C8—C7	121.3 (4)	C8—C17—H17	119.9
C17—C8—H8	119.3		

Symmetry codes: (i) −*x*+1, −*y*+1, −*z*.
